# An Overview on the Treatment of Oil Pollutants in Soil Using Synthetic and Biological Surfactant Foam and Nanoparticles

**DOI:** 10.3390/ijms24031916

**Published:** 2023-01-18

**Authors:** Kien A. Vu, Catherine N. Mulligan

**Affiliations:** 1 Department of Civil and Environmental Engineering and Earth Sciences, University of Notre Dame, Notre Dame, IN 46556, USA; 2 Department of Building, Civil and Environmental Engineering, Concordia University, Montreal, QC H3G 1M8, Canada

**Keywords:** soil remediation, oil pollutants, surfactant, biosurfactant, surfactant foam, nanoparticles, nanoparticle-stabilized surfactant foam

## Abstract

Oil-contaminated soil is one of the most concerning problems due to its potential damage to human, animals, and the environment. Nanoparticles have effectively been used to degrade oil pollution in soil in the lab and in the field for a long time. In recent years, surfactant foam and nanoparticles have shown high removal of oil pollutants from contaminated soil. This review provides an overview on the remediation of oil pollutants in soil using nanoparticles, surfactant foams, and nanoparticle-stabilized surfactant foams. In particular, the fate and transport of oil compounds in the soil, the interaction of nanoparticles and surfactant foam, the removal mechanisms of nanoparticles and various surfactant foams, the effect of some factors (e.g., soil characteristics and amount, nanoparticle properties, surfactant concentration) on remediation efficiency, and some advantages and disadvantages of these methods are evaluated. Different nanoparticles and surfactant foam can be effectively utilized for treating oil compounds in contaminated soil. The treatment efficiency is dependent on many factors. Thus, optimizing these factors in each scenario is required to achieve a high remediation rate while not causing negative effects on humans, animals, and the environment. In the future, more research on the soil types, operating cost, posttreatment process, and recycling and reuse of surfactants and nanoparticles need to be conducted.

## 1. Introduction

Oil compounds have been widely utilized as an energy source in human life and industry for a long time. In nature, they can be found in deposits or deep sediment as a result of the decomposition of dead plants and animals over many years. They mostly consist of aliphatics (e.g., alkanes, alkenes), aromatics (e.g., polycyclic aromatic hydrocarbons—PAHs), and non-hydrocarbon compounds (e.g., sulfides, pyridine, metals) [1].

Oil pollutants can be formed by exploration, production, and transformation processes [2]. After entering the soil, the interaction of oil pollutants with soil components and microorganisms may alter their properties and transport [3,4]. They continue in the soil for a long time due to the attachment or adsorption to soil components, which can harm the soil, ecosystem, or animals [5].

In recent years, the use of oil-related products has increased as a result of economic development and population growth. According to a British Petroleum report [6], global oil consumption was 5.3 million barrels per day in 2021, whereas 1.5 million barrels per day were consumed by the United States. A small amount of oil pollutants may cause serious problems for animal and human health, such as teratogenicity, cardiotoxicity, cancer, and fetus malformation [5,7]. Moreover, they are also listed in the priority pollutants category by the United States Environmental Protection Agency (USEPA) as an origin of cancer for humans [8]. The toxicity of oil pollutants to humans is strongly dependent on their specific composition, features, and contact time and level [9,10]. In addition, the presence of oil pollutants in the soil can decrease the resistance to diseases and stunted growth of plants or limit the development of soil microbes and the aquatic environment [7,11,12]. In this review, the treatment of oil pollutants in soil by nanoparticles, surfactant foams, and nanoparticle-stabilized surfactant foams is evaluated. In addition, the removal mechanisms, the effect of some factors on the treatment performance, as well as some advantages and disadvantages of these methods will also be studied.

## 2. Remediation Methods of Oil Pollutants in Soil

Different approaches have been utilized to remediate oil pollutants in soil. Some common techniques are physicochemical (e.g., surface capping, pump and treat, soil washing, soil vapor extraction, soil extraction) [13,14,15,16], chemical (e.g., stabilization, oxidation–reduction, adsorption, supercritical fluid extraction and oxidation, encapsulation) [15,17,18], biological (bioremediation, bioattenuation, biodegradation, bioventing, biosparging, biotransformation, composting) [19,20,21,22], thermal (e.g., incineration, pyrolysis) [23,24], and phytoremediation (phytostabilization, phytovolatilization, phytotransformation) methods [15,25]. Many criteria should be considered to select the optimal treatment method, such as site characteristics, oil pollutant features, soil composition and properties, remediation time and cost [15]. Generally, these common methods have many disadvantages that limit their wide application, e.g., they are not effective for removing oil pollutants adsorbed on clay-size particles (soil washing) [26,27] or high oil content (soil vapor extraction) [25]. There is also the possibility of the formation of by-products (chemical oxidation–reduction) [17]. They are not effective for clay soils and have the potential to generate more toxic by-products (biodegradation) [22]. In addition, they have high operation costs, further treatment demand for off-gases and combustion residuals (thermal treatment) [24], and long treatment time (phytoremediation) [15]. Thus, it is critical to research and develop new oil-contaminated soil remediation approaches.

### 2.1. Application of Nanoparticles for Remediating Oil Pollutants in Soil

Nanoparticles are particles with a size of less than 100 nm (or 10^−9^ m). Due to their unique characteristics, for example, small size or high specific surface area, they can be transported to complex target zones at contaminated sites [28]. Together with their simple and uniform operating conditions [29], they have been widely used for soil remediation. In contrast, long treatment times and possible formation of toxic by-products are some disadvantages of using nanoparticles for soil remediation [30].

#### 2.1.1. Effect of Nanoparticles on Soil Properties

The presence of nanoparticles decreases the soil pH, organic carbon, activity of dehydrogenase enzyme, microbial biomass transformation rate, soil bacteria, and amount of fungal colonies in the soil, reducing the soil microbial diversity [31]. Due to their magnetic attraction, nanoparticles tend to aggregate to form larger particles, lowering soil mobility and reactivity [32].

Meanwhile, the addition of nanoparticles enhances the available phosphorus in the soil. In another study, adding ZnO nanoparticles (10 mg/kg soil) reduced the soil pH after seven days and decreased the eqCO_2_ value in soil or the conversion rate of carbon sources into biomass. However, the presence of ZnO nanoparticles also enhanced the development of some bacteria in the soil, which improved the soil microbial diversity [33].

#### 2.1.2. Removal Mechanisms

Nanoparticles have been used to remediate contaminated soil under different conditions for a long time. Due to their high solvent affinity and large specific surface area, nanoparticles can easily contact oil compounds and improve their solubility, leading to a high removal rate [34,35]. The interaction of nanoparticles and other counterparts strongly depends on their types, amount, and properties [36]. Their main treatment mechanisms are adsorption (e.g., nZVI, carbon nanotubes), oxidation (e.g., manganese nanoparticles, cobalt nanoparticles), and photocatalysis (e.g., bismuth nanocomposite, BiPO_4_-based photocatalysts) applications [37,38]. Oil pollutants can be removed from contaminated soil by adsorption on the nanoparticle’s surface via π–π and van der Waals interactions [18,39,40]. Nonetheless, the potential aggregation of nanoparticles, which can decrease the surface area and active sites of nanoparticles and reduce the treatment efficiency, is one of the most significant disadvantages of this method.

In the oxidation method, the oil pollutants can be reduced into less toxic or non-toxic compounds, such as CO_2_ and H_2_O, by Fenton-like reactions [35,41,42]. This method involves the degradation of oil pollutants by reactive oxygen species (ROS), which are formed via the reaction of iron oxides with H_2_O_2_, UV light, or under ultrasound [43]. In particular, the generation of ROS such as hydroxyl radicals (HO**^.^**) or hydroperoxyl (HO_2_**^.^**), may degrade oil pollutants to form final products, such as CO_2_ and H_2_O, as follows [44]:

Fe^2+^ + H_2_O_2_ → Fe^3+^ + OH^−^ + HO (1)

Fe^3+^ + H_2_O_2_ → Fe(OOH)^2+^ + H^+^ (2)

Fe(OOH)^2+^ → Fe^2+^ + HO_2_ (3)

H_2_O_2_ + HO**^.^** → HO_2_**^.^** + H_2_O (4)

Fe^3+^ + HO_2_**^.^**→ Fe^2+^ + H^+^ + O_2_ (5)

ROS + oil pollutants → CO_2_ + H_2_O (6)

This method is simple, cheap, effective for various organic pollutants in soil, and safe for the environment and human health. However, the potential toxicity of intermediates and slow treatment efficiency are some disadvantages that should be considered for this approach. Moreover, its treatment rate is influenced by concentrations of H_2_O_2_, the dosage of iron oxides, power and time of UV light and ultrasound, pH, and temperature [44].

**Figure 1:**
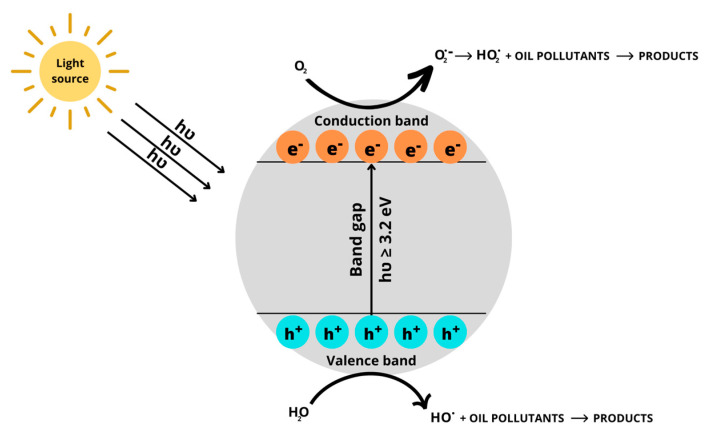
Treatment of oil pollutants by photocatalytic activity of TiO_2_ nanoparticles.

In the photocatalysis method, oil pollutants are degraded into mostly CO_2_ and H_2_O by reactive oxygen species, such as HO**^.^** or superoxide anions (O_2_**^.^**), formed under the activation of light and semiconductors such as TiO_2_ or ZnO [45]. In particular, under the illumination of a light source, such as ultraviolet (UV) or sunlight, electrons from the valence band in TiO_2_ nanoparticles will be activated and jump to the conduction band, leaving behind some holes—h^+^ (Figure 1). These h^+^ and e^−^ may react with H_2_O and O_2_ in the atmosphere to generate ROS, which will degrade oil pollutants into less toxic or non-toxic products. The advantages of photocatalytic techniques include high treatment efficiency, clean technology, high stability, no formation of toxic by-products, and low toxicity. Meanwhile, the high energy cost, quick recombination rate of ROS, and unavailability of pollutants deep in the soil are some disadvantages of this method. The treatment efficiency by photocatalysis methods is strongly dependent on various factors, such as temperature, soil particle size and type, soil thickness, humic acid, light source and time, and characteristics of oil compounds in soil [46].

#### 2.1.3. Treatment of Oil Pollutants in Soil by Nanoparticles

Various nanoparticle types have been successfully utilized to remove different oil pollutants from contaminated soil. Carbon nanotubes (CNTs) were effectively utilized for adsorbing PAHs [47] or dichloro-diphenyl-trichloroethane (DDT) in the natural soil, whereas 56% of DDT was degraded by nZVI after 7-day treatment [48]. Bentonite clay combined with nZVI removed PCBs from soil-sorbed PCBs 10 times more than only nZVI [49]. Furthermore, the addition of ethanol increased PCB desorption and enhanced the contact between PCBs and nZVI, leading to 50% higher treatment efficiency.

Iron nano-oxide particles removed 99% pyrene in contaminated soils via a Fenton oxidation reaction with hydrogen peroxide (H_2_O_2_) [50]. Karam et al. [51] showed a high degradation rate of anthracene using nano-TiO_2_-photocatalysts. Furthermore, PAHs were productively treated by different nanoparticles, such as gold nanoparticles [52], iron hexacyanoferrate nanoparticles [53], ZrO_2_ nanoparticles [54], nano Fe^3+^-montmorillonite [55], nano anatase TiO_2_, [56], ZnO nanoparticles [57], Ti/ZnO-Cr_2_O_3_ nanocomposite [58], Fe_3_O_4_ nanoparticles [59], TiO_2_-graphene nanocomposites [45], Fe/Cu bimetallic nanoparticles [38,60]. More oil-contaminated soil treatment methods using nanoparticles are shown in Table 1.

In other research articles, nanoparticles have been combined with microorganisms to remove oil pollutants in soil. The presence of nanoparticles might have improved microbial metabolism and microbial enzymes, which increased the treatment efficiency of toxic organic contaminants [77]. The combination of iron magnetic nanoparticles and *Bacillus* spp. degraded up to 89.7% atrazine in soil [78]. Bebić et al. [79] indicated the degradation of lindane up to 68.3% using silica nanoparticles and *Myceliophthora thermophila* at pH 5.0 in 40 min.

## 3. Surfactants

### 3.1. Surfactant Characteristics

**Figure 2:**
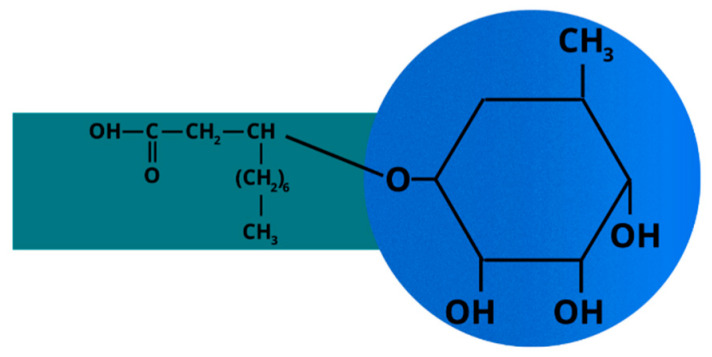
Structure of rhamnolipid biosurfactant with hydrophobic tail (green) and hydrophilic head (blue).

Surfactants are amphiphilic compounds with hydrophilic heads and hydrophobic tails (Figure 2). They have generally been employed in human life or industry as detergents, adhesives, or foaming agents [80]. They may reduce the surface tension or interfacial tension of water, enhancing the solubility of hydrophobic compounds. Low toxicity and high biodegradability also contribute to their wide use in environmental applications.

Surfactants include three main types: nonionic, anionic, and cationic surfactants. Nonionic surfactants are defined as surfactants with uncharged hydrophilic head groups [81]. In nonionic surfactants, hydrophilic and hydrophobic groups are generally polyoxyethylene and linear or branched alkanes. Due to their nonionized property in the aqueous phase, the hydrophilic groups of some nonionic surfactants are inert to the acids and alkalis [80]. The strong hydrogen bonds between hydrophilic groups and water make them dissolvable in an aqueous solution. Their uncharged and ionized status also makes them easy to agglomerate to generate micelles due to no repulsive force between monomers [82].

The head of anionic surfactants is negatively charged hydrophilic groups. Their head and tail components are typically sodium and phosphate ions, respectively [14]. Due to the formation of ionic bonds with water, their anionic head component can dissolve in the aqueous solution. Therefore, it is hard to separate anionic surfactants from an aqueous solution. Due to the repulsive force between monomers, it is easier to aggregate anionic surfactants to form micelles than nonionic surfactants [83]. However, as a result of the electrostatic repulsion force, the negatively charged head limits their adsorption on negatively charged soil. Thus, their treatment efficiency of oil pollutants in the soil is typically better than nonionic surfactants on the lab and field scale [84,85].

Cationic surfactants can also dissolve in the aqueous phase. Due to their high toxicity to the environment and significant adsorption in soil particles, they are not widely used to treat oil pollutants in soil [86]. They are generally combined with other techniques, such as electrokinetic or bioremediation, for soil remediation [87,88].

Biosurfactants are biologically formed from plants and microorganisms. The hydrophilic groups in their component are generally derivatives of polysaccharides, amino acids, or peptides. Their hydrophobic groups are peptides or fatty acids [14]. Most biosurfactants are nonionic and anionic surfactants [86]. Due to their adsorption or replacement of water or oil molecules at the interface of oil/liquid or solid/liquid phases, biosurfactants can decrease the surface tension or interfacial tension of these phases, which will release the oil molecules out of the contaminated soil [89]. Biosurfactants can alter the wettability of soil particles via the adsorption of the hydrophobic component on the soil surface and the interaction of the hydrophilic component with the water phase [22]. The repulsive behavior between biosurfactant head and soil particle also improves the separation of oil pollutants from soil. In summary, the remediation mechanisms of oil pollutants in soil by biosurfactants are mainly interfacial tension reduction, emulsification, and change in soil wettability. Therefore, biosurfactants have been widely used for the remediation of oil pollutants in contaminated soil [14,40,86]. The use of some surfactants for removing oil from the contaminated soil is shown in Table 2.

Two of the most common biosurfactant types that have been effectively used to remove oil compounds from the soil are rhamnolipid and sophorolipid. Rhamnolipid biosurfactants (Figure 2) are produced from *Pseudomonas aeruginosa*. They can biodegrade different organic compounds in soil, such as hexadecane or petroleum hydrocarbons [80]. Sophorolipid biosurfactants are biologically generated from *Candida* yeasts. They are commonly used as cosmetic or moisturizer ingredients [94].

### 3.2. Critical Micelle Concentration of Surfactant

**Figure 3:**
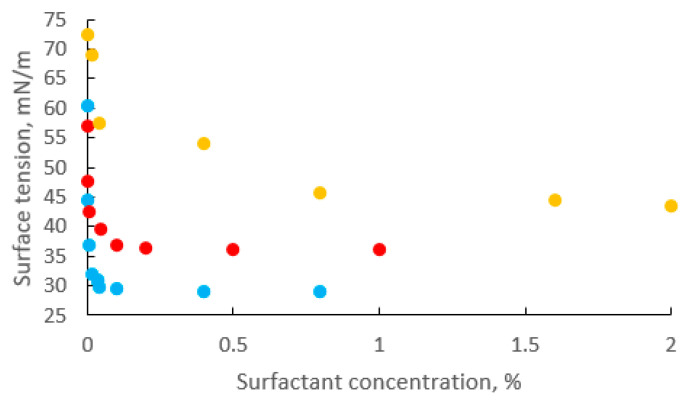
Surface tension values at various concentrations of rhamnolipid biosurfactant (blue), sophorolipid biosurfactant (red), and Ultraplex surfactant (yellow).

The efficiency of surfactants relies on their capacity to lower the surface tension or interfacial tension of water, which relates to the critical micelle concentration (CMC) value. In an aqueous solution, CMC is defined as the lowest surfactant concentration to form micelle [94]. Once the surfactant concentration is higher than the CMC, the hydrophobic component will agglomerate, while the hydrophilic part will react with the aqueous portion of external compounds, which is called the solubilization process. In contrast, lower surfactant concentration than CMC will decrease the surface tension and interfacial tension of soil/water and oil/water phases, leading to a decrease in capillary force between oil and soil [95]. However, too low a surfactant concentration may be ineffective for soil remediation due to adsorption on soil particles. Therefore, solubilization is preferred to mobilization to obtain higher soil remediation efficiency [14]. Figure 3 shows the CMC values of some common surfactants. The CMC values of rhamnolipid biosurfactant, sophorolipid biosurfactant, and Ultraplex surfactant are 0.04%, 0.1%, and 0.8%, respectively.

### 3.3. Degradability of Surfactants

The biodegradability of surfactant solution can be determined in various ways. In an experiment developed by Franzetti et al. [96], the oxygen consumption of Tween 80 surfactant and microbes in a biochemical oxygen demand (BOD) bottle was more than in an aerosol MA+80 surfactant. Therefore, the biodegradability of Tween 80 was higher than aerosol MA+80, which reflected its lower potential harm to the soil. The biodegradability of surfactants can be measured through soil microbial growth [97]. In particular, soil microbes can use surfactants as carbon sources for their growth. Therefore, higher soil microbial growth will represent greater biodegradability of surfactant in the soil. Highly degradable and low-toxicity surfactants are recommended for soil remediation due to having fewer negative environmental effects [98]. The degradability of some surfactants is shown in Table 3.

## 4. Surfactant Foam

Foam technology involves the dispersion of gas bubbles into a smaller liquid volume. Foams are generated by the small thickness of liquid between bubbles. Foams can be morphologically categorized as kugelschaum and polyederschaum, which contain spherical and polyhedral bubbles, respectively [110]. The main component of foam is gas; thus its bulk density is closer to gas than surfactant. The surface area of a certain amount of foam is significant due to its low density. The generation and disappearance of foam involve different processes, such as the movement of liquid from interfacial thin films, the dispersion of liquid across the foam-generating column, the diffusion of gas from small to large bubbles, and the seepage of liquid from the foam matrix [111]. If the surfactant concentration increases to approximately the CMC value, more foam will be generated. When surfactant molecules are present at the interface, the hydrophobic part is driven away from the solution due to the strong repulsion forces with water molecules, which will cause favorable adsorption and reduce the interfacial tension at the liquid–vapor phases [112].

### 4.1. Surfactant Foam Properties

Two critical characteristics representing aqueous foam are foamability and foam stability. Foamability is the surfactant ability to generate foam and is determined by the volume of foam produced (Equation (7)). Foam stability represents the tendency of foam to protect against bubble collapse [13]. The type and features of surfactant and gas, the characteristics of the soil, and the stability of oil/liquid/air interface play a critical role in foam stability [113]. In particular, increasing the hydrophobic chain length of a surfactant or decreasing the hydrocarbon chain length of oil pollutants may improve foam stability [114]. The existence of oil compounds in the soil during oil extraction or remediation processes may decrease foam stability due to drainage out of the foam film and the formation of the oil phase in the soil column. In addition, the adherence of oil compounds on the foam–film interface may decrease foam stability, resulting in a shorter foam column [115].

(7) $$Foam\;quality\;\left(\%\right)=\frac{Total\;gas\;volume}{Total\;foam\;volume}*100\%$$

Foam effective viscosity in a column also plays a critical role in the foam stability due to its relationship with soil permeability change [116]. In particular, it may characterize the foam flow through the porous medium under various conditions. Foam viscosity can follow the single-phase Darcy equation (Equation (8)). Chowdiah et al. [116] showed an increased foam effective viscosity with soil permeability. Meanwhile, the foam mobility did not change much with different soil permeability values, which suggested an option to prevent fluid leakage into channels with high soil permeability.

(8) $$\mu_{foam}=\frac{kA}{q_{foam}}*\frac{\Delta P}{\Delta L}$$

where *µ_foam_* is foam effective viscosity, kg/cm·s

*k* is soil permeability, cm/s

*A* is column cross-section area, cm^2^

*q_foam_* is flow rate, cm^3^/s

∆*P* is pressure drop, kPa

∆*L* is column length, cm

The resistance factor for the flow of foam in porous media also influences the remediation performance. It is defined as the ratio of pressure change in the soil layer and water flushing to achieve pressure change at a steady state in foam flushing [117]. It was found that an increase in the resistance factor would improve foam stability, resulting in higher remediation efficiency [118]. Pressure gradient (the pressure ratio inside the column) and column length may affect foam transport, quality, and stability. An optimal pressure gradient should be maintained during foam flushing to accommodate the foam bubbles for a long time [119].

### 4.2. Use of Surfactant Foam for Soil Remediation

#### 4.2.1. Removal Mechanism of Contaminants in Soil by Surfactant Foam

Soil remediation by surfactant foam includes three steps: the flow of foam throughout the soil layer, desorption of contaminant from the soil, and transfer of contaminant out of the soil. The first step plays a vital role in the remediation efficiency [120]. Interaction of the oil phase and surfactant foam may happen in two ways: oil compounds can invade the surfactant foam, or surfactant foam can slide over water molecules containing the oil [121]. The foam mobility through the soil media is dependent on the formation and collapse of the bubble, capillary pressure, and channeling properties [122,123]. Osei-Bonsu et al. [124] exhibited that the transport of stable foam might reduce gas flow in the soil layer, decreasing gas mobility and permeability. In other words, a highly stable foam in a contaminated soil layer will provide higher soil remediation efficiency than a lowly stable foam. In another in situ remediation technique, stable foam was injected horizontally into a contaminated soil zone via injection well flows. The contaminant was removed through desorption from the soil and adherence to the foam processes. The movement of foam in porous media strongly depends on the foam stability. In general, foam stability is enhanced with increased pressure alternation by water flushing across the soil layer, resulting in higher treatment efficiency [118]. Therefore, an optimal pressure gradient is necessary to allow surfactant foam to pass through the soil layer.

The main removal mechanism of oil compounds by surfactant foam includes solubilization and mobilization, which happens at surfactant concentrations above and below the CMC value, respectively [40]. The treatment process of oil pollutants from soil may be caused by the increase in solubilizing capacity of oil compounds or the reduction of surface tension between oil and the aqueous phase of surfactant foam, leading to the mobilization of oil compounds [125].

Surfactant foam can be collapsed due to the diffusion and extension of oil compounds into the gas–liquid interface of bubbles on the soil surface [114]. The formation and development of surfactant foam may also be limited by the adherence of salts (e.g., calcium or sodium) and chelating agents (e.g., ethylenediaminetetraacetic acid—EDTA) on the foam surface that cause a decrease in foam volume [126]. However, a suitable surfactant type and foam stabilizing agent can stabilize the surfactant foam and avoid these issues.

#### 4.2.2. Use of Surfactant Foam for Remediation of Oil Pollutants from Soil

- Nonionic surfactant foam

Nonionic surfactant foam has been employed for removing contaminants from soil. The treatment performance of n-pentadecane from a contaminated column by nonionic surfactant Triton X-100 (85% at the surfactant concentration of 2000 mgL^−1^) was higher than surfactant solution (26% at the same surfactant concentration) [127]. This result was similar to Parnian and Ayatollah [128], where Triton X-100 surfactant foam demonstrated higher remediation effectiveness of diesel from clayey loam soil in a column study than Triton X-100 surfactant solution at the same surfactant concentration. In the presence of 0.1% polydimethylsiloxane oil, the most common antifoaming and defoaming agent to control the foam height, 53.48% and 75.92% of 16 polycyclic aromatic hydrocarbons (PAHs) were removed after 5 min and 30 min washing time, respectively [129]. These removal efficiencies were higher than Triton X-100 (44.12% and 67.28%), which reflected the effectiveness of using proper defoamer in the remediation of PAHs from contaminated soil. Mulligan and Eftekhari indicated a 85% removal rate of pentachlorophenol from fine sand using 5000 mgL^−1^ Triton X-100 foam [123]. In another study, Triton X-100 foam removed 94% of the transformer oil from quartz sand and 85% of polychlorinated biphenyls (PCBs) from coarse sand [117]. Tween 80 foam remediated 87% diesel oil from sandy soil, suggesting an inexpensive and effective method for soil washing [130]. At low temperature (6 °C), spraying aqueous Tween 80 nonionic surfactant foam removed 73.7% of total petroleum hydrocarbon (TPH) from contaminated soil. In addition, the optimal temperature for microbes to biodegrade contaminants was also accommodated by the surfactant foam [20]. Maire et al. [131] indicated a maximum 95% removal efficiency of dense nonaqueous phase liquid (DNAPL) from contaminated soil using Tergitol nonionic surfactant foam, which is more effective and less expensive than using a surfactant solution only.

- Anionic surfactant foam

**Figure 4:**
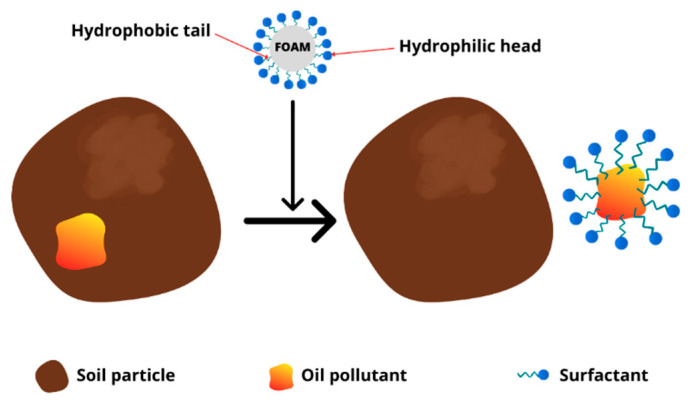
Use of surfactant foam to remove organic contaminants in soil.

Anionic surfactant foam has been effectively used for soil remediation (Figure 4). The anionic surfactant foam created by Steol CS-330 and SDS removed 60% and 75% of the trichloroethylene (TCE) and TCE-DNAPL from the contaminated sand column, respectively [132]. In another paper, the removal rate of diesel oil from contaminated sandy soils by anionic surfactant SDS foam (88%) was much higher than by surfactant solution only (35%) [133]. Similarly, surfactant SDS foam-spraying and surfactant SDS microfoam demonstrated a maximum 73.7% and 62% removal productivity of diesel oil from sandy soil, respectively [20,134]. Meanwhile, using nitrogen gas, an anionic surfactant sodium lauryl ether sulfate (SLES) foam removed 68% TCE from soil sediment. Wang and Chen [135] indicated a maximum 76% remediation performance of PCBs from quartz sand after a 30-pore volume flushing by SDS foam. Using surfactant-stabilized foams might improve their transport in the soil layer and help to remove 95% DNAPL from contaminated soil after four days [136].

In other studies, anionic surfactant foam has been effectively used to remediate various soil pollutants (Table 4).

- Effect of various factors on the remediation of oil pollutants in soil by surfactant foam

The treatment performance of oil pollutants relies on the amount and properties of specific oil compounds. Sihag et al. [138] showed a relationship between oil structure and concentration and the capacity of soil microbes to break down oil pollutants in soil. Due to their higher water solubility and bioavailability, it is easier to degrade the intermediate-chain alkanes (C_10_-C_25_) than long-chain alkanes (C_25_-C_40_). The degradation performance of linear chain alkanes is higher than branched alkanes and cycloalkanes. The treatment efficiency of complex and high oil concentrations is low due to their potential toxicity, which reduces the growth and activity of soil microbes. Similarly, the small solubility of high-molecular-weight oil compounds may reduce their interaction with soil microbes, resulting in low treatment efficiency [139].

Soil characteristics, such as pH, humidity, temperature, nutrient availability, or soil component, may influence the development of soil microorganisms and play a key role in the soil remediation rate. In particular, high temperature may increase oil solubility, diffusion, and bioavailability while reducing oil viscosity, leading to higher treatment efficiency [140]. The optimal temperature for soil remediation is about 25–35 °C [23,40]. Soil structure also influences the fate and transport of oil pollutants in soil, altering their treatment efficiency in soil [141].

The optimal soil pH and C:P:N ratio, which represents the nutrient amount, should be maintained to improve the microbial growth and bioavailability of oil pollutants, leading to higher removal effectiveness [19]. Limited nutrient content may inhibit the growth of hydrocarbon-degrading microorganisms, which restricts the rate of oil pollutant degradation and reduces the bioremediation performance. Meanwhile, a too low or too high a nutrient amount may limit the growth and activity of soil microorganisms, leading to a low degradation rate.

The transport and availability of oxygen may affect the oil treatment efficiency. Highly dissolved oxygen content in soil can improve the growth and activity of soil microorganisms, leading to a high degradation rate. In particular, high oxygen concentration may enhance the activity of oxygenase, favoring the respiration process and increasing the oil remediation performance. Oxygen availability in soil strongly relies on soil type and moisture content [138].

Soil organic matter may control the soil remediation rate by inhibiting the interaction of soil particles and oil pollutants [142]. High soil organic matter levels can increase the partition of oil pollutants into soil fractions, which decreases the sorption rate of oil pollutants and reduces soil remediation efficiency [143]. The remediation rate of oil pollutants is high at the early treatment stage due to the highly bioavailable oil compounds. After that, oil pollutants are partitioned into soil organic matter, which limits their bioavailability and leads to low treatment efficiency [144]. For soil with low organic matter, the oil pollutants may penetrate small pores on the soil surface, which reduces their bioavailability by microorganisms, leading to lower removal efficiency [145]. Therefore, the treatment rate of oil pollutants in freshly contaminated soil is generally higher than in aged soil. Moreover, fine soil with high organic matter can create favorable conditions for the growth of soil bacteria, leading to a high biodegradation performance of oil pollutants [141,146].

## 5. Remediation of Oil Pollutants in Soil by Surfactant Foam/Nanoparticle Mixture

### 5.1. Interaction of Surfactant Foam and Nanoparticles

**Figure 5:**
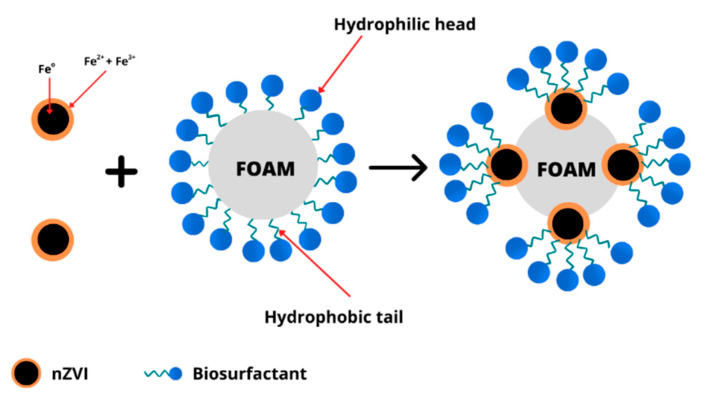
Interaction of surfactant foam and nZVIs.

The existence of contaminants may decrease the spreading velocity of aqueous foam in soil layers [147]. In this case, the foam interaction with soil contaminants can be represented by the dimensionless Lamela number [148]. Hence, nanoparticles can stabilize the surfactant foam (Figure 5), enhancing their movement in the unsaturated soil zone [149]. The foam stabilization by nanoparticles involves the agglomeration of nanoparticles at the oil–water interface to create a thick layer that may hinder foam aggregation [150]. In particular, the generation of nanoparticle monolayers or adjacent nanoparticle bilayers may cause the stabilization of liquid films in the foam [151,152]. The attachment of colloidal nanoparticles at the gas–liquid or liquid–liquid interfaces of foam may decrease bubble breakage, contributing to foam stabilization [153,154]. For example, the foam formation by silica nanoparticles and SDS surfactant was 10 times more stable than that by only SDS surfactant due to the attachment of SDS molecules, which lowered the silica nanoparticle surface charge [154]. Li and Prigiobbe [155] showed a similar result, where high foam quality was formed by cationic surfactant and silica nanoparticles under N_2_-gas. The generation mechanism of anionic surfactant foam in porous media with or without nanoparticles is similar [156]. In another study, the mixture of hydrophobic fine particles and surfactant was also proven to improve the bubble combination efficiency and reduce the foam stability [157]. The reduction in nanoparticle retention due to the decrease of surface tension at the liquid–gas interfaces may influence foam stability [158,159]. The gas used for foam generation may also influence the transport of surfactant foam–nanoparticle mixture through the soil, affecting the remediation efficiency of oil pollutants [160].

Nanoparticles can also stabilize and enhance foam transport in the unsaturated zone of the soil, leading to higher soil treatment effectiveness [160]. The accumulation of nanoparticles at the oil–water interface may generate particle monolayers or bilayers, which limit bubble breakage and stabilize the surfactant foam [150]. In addition, the adsorption of colloidal nanoparticles at gas–liquid or liquid–liquid interfaces may also contribute to foam stability [153]. The foam produced by silica nanoparticles and SDS surfactant is ten times more stable than by only SDS surfactant. This is due to the adsorption of SDS surfactant foam on the nanoparticle surface lowering the surface charge, which promotes the capacity to adsorb more uncharged nanoparticles on the foam surface [154]. The foam stability produced by hydrophobic fine particles and surfactant is much lower than by surfactant only due to the development of bubble coalescence [157].

### 5.2. Use of Surfactant Foam–Nanoparticle Mixture in Soil Remediation

The effect of colloidal particles on foam formation, stability, and prevention has been studied for a long time [161]. Due to their ability to enhance foam stability, nanoparticles have been effectively used for oil recovery [162] or soil remediation [38,40,163]. The viscoelastic layer was enhanced by attaching 50% silica nanoparticles to the interface, which hindered the collapse of the bubble and improved the foam stability up to 23 h [164]. In another study, the half-life of SDBS surfactant foam after adding silica nanoparticles was double that of only SDBS surfactant foam due to the development of foam stability [165]. The presence of silica nanoparticles in a sand column also decreased the hydraulic conductivity of CTAB surfactant foam, which enhanced the foam stability after 17 days and led to higher isolation efficiency of the contaminant in soil [166].

The transport of nanoparticles in soil was improved by combining them with surfactant foam. Surfactant foam may stabilize the nanoparticle suspension and prevent them from aggregating in an aqueous solution, which decreases the nanoparticle retention on the soil surface and enhances the movement of nanoparticles in the soil. For example, 1% SLES surfactant foam delivered 100% nZVI in the soil vadose zone, leading to higher removal efficiency of the soil contaminants [167]. Shen et al. [168] reported that the transport of nZVI in the soil subsurface was significantly improved with foam generated by different surfactants, such as SDS, TW20, TW80, TX100, leading to higher remediation efficiency in the vadose zone. The film breakage of foam was reduced with the addition of nZVI, which enhanced the microsphere transport in soil and led to better treatment effectiveness. The same results were reported in other papers, where the remediation rate of oil contaminants was 78–99% by foam-stabilized nanoparticles under various environmental conditions [169,170].

Different nanoparticles and surfactant foam have been successfully utilized to remediate various soil contaminants (Table 5). The treatment efficiency of diesel oil in contaminated soil was 78% and 95% using TW20 surfactant foam–SiO_2_ nanoparticles and SDS surfactant foam–SiO_2_ nanoparticles, respectively, which was higher than using only surfactant solution (42%) [169,170]. Singh and Mohanty [171] reported higher recovery of crude oil from sandstone core by alkyl polyglucoside (APG) surfactant foam–silica nanoparticles (54%) than by surfactant foam (25%). only This result was similar to previous studies using SDS surfactant foam–silica nanoparticles [159] and TW20 surfactant foam–nano-zerovalent iron [167].

Using a surfactant foam–nanoparticle combination for soil remediation is less common than surfactant solutions only or surfactant foams. However, due to the synergistic effect of nanoparticles and surfactant foam, the soil remediation efficiency by this mixture is surpassed by only surfactant or surfactant foam at the same concentration [170]. With other advantages, such as simplicity and effectiveness for various soil contaminants, applying a surfactant foam–nanoparticle mixture can become a productive method for soil remediation in the future.

**Figure 6:**
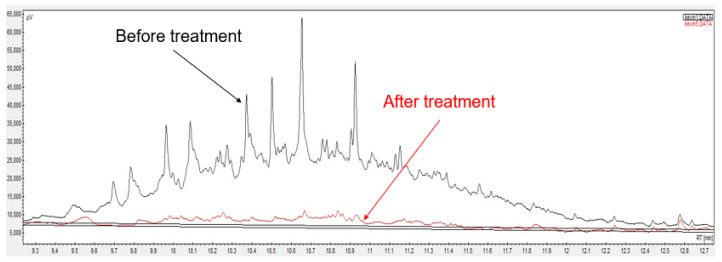
Peaks of oil pollutants before (black) and after (red) treatment by rhamnolipid biosurfactant foam and nZVI.

A surfactant foam–nanoparticle mixture has been employed for soil remediation in the field. According to Quinn et al. [176], the treatment performance of TCE from contaminated soil was remarkably enhanced (up to 100%) after five months. In another article, Zhao et al. [177] pointed out that the degradation rate of chlorinated volatile organic compounds (CVOCs) and perchloroethylene (PCE) in field sites using a surfactant–corn oil–nZVI mixture after 2.5 years was 86% and 93%, respectively. He et al. [178] showed that 88% TCE was removed using carboxymethyl cellulose and Fe/Pd nanoparticle mixture after 596 days. Moreover, the presence of hydrogen improved the remediation performance. Bennett et al. [179] pointed out that the chlorinated ethenes at an aerospace facility were rapidly degraded by applying a carboxymethyl cellulose and Fe/Pd nanoparticle combination. The reduction in oil concentration from contaminated soil using biosurfactant foam–nanoparticle mixture has been shown by gas chromatography (Figure 6).

### 5.3. Effect of Some Factors on Soil Remediation Performance by Surfactant Foam–Nanoparticles

#### 5.3.1. Effect of Environmental Conditions

The environmental conditions (or weather) are one of the most vital factors affecting the remediation efficiency of surfactant foam–nanoparticles. The weather may change the toxicity and biodegradability of surfactant foam, which alters the properties of the surfactant foam–nanoparticle mixture [103]. Toxicity and biodegradability are possible adverse effects of surfactant foam on the soil and the potential influence of soil microorganisms on the surfactant foam, respectively [108]. Due to the low biodegradability of most chemical surfactant foams used for soil remediation, combining these surfactant foams with nanoparticles may adsorb on the soil surface and harm the soil properties and soil microorganisms [180].

The adsorption of these surfactant foams, especially nonionic surfactant foam, on the soil surface can generate aggregation, which will alter the soil hydrophobicity, reduce soil retention, and cause toxicity to the soil [181]. The toxicity to the soil is more serious due to the potential absorption of surfactant foam into the plant roots, which can decrease crop growth and yield. In addition, the surfactant foam can break the cellular membrane, interact with lipids and proteins, and harm the soil microorganisms. The potential toxicity of some surfactants on soil microbes has been indicated in previous studies [182,183]. Therefore, biosurfactant foam, biologically produced from the microbial population, is suggested for soil remediation [34,184].

#### 5.3.2. Effect of Soil Characteristics

Soil type and particle size can affect the remediation rate. The smaller the soil particle size, the higher porosity and stronger bonds with oil pollutants, which will decrease the soil wettability and lead to lower treatment effectiveness. The remediation of motor oil from clay soil (soil porosity of 68.7%) was found to be lower than from desert soil and coastal soil (soil porosity of 42.5% and 37.5%) [86]. In another study, the removal performance of PCBs in clay soil was lower than in sandy soil due to the smaller desorption of PCBs in clay soil [17].

The presence of organic matter in soil components creates more competitive factors with oil pollutants in the mixture, which will inhibit and limit the removal efficiency of oil pollutants [185]. The bond of organic matter molecules to the nanoparticle surface may generate a film that prevents the mass and electron transfer rate, resulting in a lower remediation percentage [16]. Soil type also changes the activation energy connecting oil pollutants and soil surface, thus influencing the oil remediation efficiency [186]. In particular, the binding of adsorbed oil compounds and soil particles may cause clogging and block the available pores, limiting the transport of flow through the pores and decreasing the removal effectiveness [91]. A reduction in treatment efficiency was also observed by adding some salts representing the ionic strength in soil components [40]. Consequently, treatment of oil compounds from contaminated soil greatly relies on the soil characteristics.

Soil pH also plays a critical role in the treatment efficiency of oil pollutants. Mańko et al. showed a change in CMC value and micelle generation because of the pH effect on the surface and interfacial tension of surfactant molecules, which will alter the oil treatment performance [165,187,188,189]. At low pH, more H^+^ ions are present, which makes the soil surface more positively charged, leading to a higher reduction of oil pollutants from the soil [190].

#### 5.3.3. Effect of Nanoparticle Properties

The surface area of nanoparticles plays a vital role in the treatment effectiveness of surfactant foam–nanoparticle mixtures. The higher the surface area of nanoparticles, the greater the remediation rate. The larger surface area will lead to more interaction between nanoparticles and oil pollutants. In other words, more oil compounds may be adsorbed, complexed, or reduced on the nanoparticle surface, resulting in higher treatment efficiency [191]. The high specific area also increases the agglomeration of nanoparticles due to their magnetic attraction, which may reduce their reactivity and mobility in soil, subsequently leading to lower remediation efficiency. However, the presence of surfactant foam may act as a stabilizer and inhibit nanoparticle aggregation [34]. The use of 20 nm Fe/Cu nanoparticles showed higher treatment efficiency of oil pollutants in soil than 200 nm Fe/Cu nanoparticles [40].

**Figure 7:**
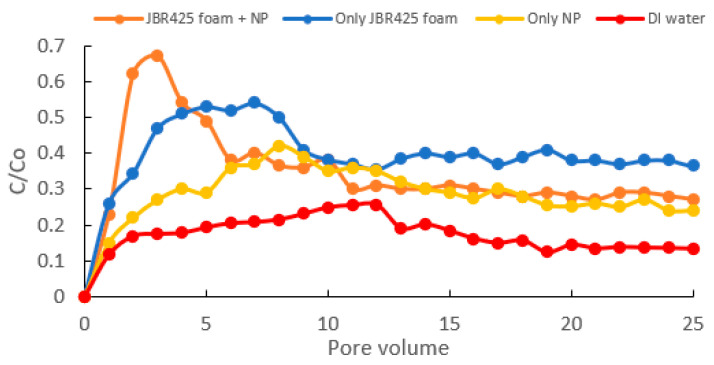
Oil treatment rate (C/Co) in column experiments using JBR425 rhamnolipid biosurfactant foam and Fe/Cu nanoparticles (NP) under various conditions.

The interaction of nanoparticles and hydrophilic components of surfactant foam may prevent surfactant foam collapse and enhance foam stability and quality [10,40,192]. The repulsive electrostatic force between nanoparticles and surrounding liquid is improved due to the adsorption of surfactant molecules on the solid–liquid interface, which will lower the surface tension of the mixture, resulting in a change in remediation rate [193]. If the number of nanoparticles exceeds the threshold value, more surfactant molecules will be attracted to the solid–liquid interface. Therefore, fewer surfactant molecules appear at the gas–liquid interface, reducing the cohesive force between surfactant molecules. Consequently, the surfactant foam will collapse, and treatment efficiency will decrease [192]. The increase in nanoparticle quantity may also improve the attractive van der Waals force, decreasing the interfacial tension of surfactant and oil pollutants and affecting the soil remediation rate [194]. The role of biosurfactant foam and nanoparticles on the remediation of oil pollutants in soil is shown in Figure 7, where the oil treatment efficiency by biosurfactant foam/nanoparticle mixture is higher than only biosurfactant foam and only nanoparticles.

#### 5.3.4. Effect of Surfactant Concentration

The presence of surfactant foam may prevent nanoparticle aggregation, which increases the delivery and transport of nanoparticles in soil, leading to higher treatment efficiency [34,84]. Surfactant concentration changes the CMC value of the mixture, which may alter and surface and interfacial tension of oil pollutants, resulting in a change in oil treatment efficiency. Moreover, foam quality and stability greatly depend on the surfactant concentration. At pH 7, the combination of 2 vol% rhamnolipid and 2 wt% Fe/Cu nanoparticles displayed high foam stability and quality, leading to a better remediation rate of oil pollutants in soil [34]. Therefore, a suitable amount of surfactant can generate high foam quality, improving the aggregation of nanoparticles at the interface and increasing the interaction of a surfactant foam–nanoparticle mixture with oil pollutants, resulting in high effectiveness.

### 5.4. Limitations

Nanoparticles can penetrate organisms through ingestion or inhalation and cause some negative effects. The toxicity of nanoparticles to humans, animals, and soil microorganisms has raised some public concerns [195,196]. Some adverse effects are cell membrane damage, respiration interference, and DNA oxidative damage [197,198]. When nanoparticles enter the cell, they may concentrate at the cell membrane and increase their concentration on the cell surface. Some nanoparticles, such as nZVI or nano-iron oxide, can react with hydrogen peroxide on the cell surface to generate ROS, damaging the cell membrane [199]. In addition, nanoparticles may precipitate on the cell surface through the interaction with lipoteichoic acids in the cell wall, which will block the pores on the outer cell membrane, prevent nutrient transformation, and lead to the death of the cell [200].

However, the potential toxicity of nanoparticles is still controversial and needs more research. Vanzetto and Thome [201] found that nZVI caused no negative effect on the development of bacteria (*Bacillus* and *P. aeruginosa*) in pentachlorophenol-contaminated soil during the nanoremediation process. No major change in temperature, electrical conductivity, pH, and humidity of soil was observed after 90 days. Fajardo et al. [202] found no cytotoxicity on *Klebsiella planticola* bacteria in soil by the high concentration of nZVI. Nanoparticles have little or no adverse influence on the growth of different fungi, such as *Trametes versicolor* and *Aspergillus versicolor*. In other studies, nanoparticles caused no significant toxicity on different bacteria, such as *P. stutzeri*, *Klebsiella oxytoca, P. putida*, or *Escherichia coli* under various incubation conditions [199,203,204,205]. The resistance mechanisms of bacteria or fungi are mainly due to the limitation of nanoparticle adsorption into the cell by some cell wall components, such as intracellular antioxidants, which decreases the adverse effects of nanoparticles [193,194]. Chitin cell walls also play a critical role in the low adsorption of nanoparticles, leading to their high resistance to nanoparticles [189,198].

## 6. Conclusions and Future Research

Oil pollutants in soil have become a primary environmental problem due to their wide application and toxicity. They can enter and contaminate the soil from oil production and improper disposal, which may cause adverse effects on human health and the environment. Various nanoparticles and surfactant foams have been effectively used to remediate oil pollutants in the soil on the lab and field scale. The main treatment mechanisms are adsorption and reduction (for nanoparticles) and solubilization and mobilization (for surfactant foam). Among different surfactant types, biosurfactants can produce more stable foam. With the low toxicity and high biodegradability, it is recommended to use the nanoparticle–surfactant foam mixture for the treatment of oil pollutants in the soil. The addition of nanoparticles was found to greatly improve foam stability, leading to a higher remediation rate.

The oil treatment performance by nanoparticle-stabilized surfactant foam relies on various factors, such as environmental conditions, soil properties and amount, nanoparticle dosage, and surfactant concentration. Optimizing these factors in each scenario will help to obtain high treatment efficiency with low operating costs while not causing negative effects on the environment.

Some disadvantages, such as the potential toxicity of nanoparticles, the stability of the mixture, or fast surfactant foam desorption into water from the soil, may limit their wide application. More studies on these factors are necessary to evaluate their influence on soil remediation, especially in long-term and full-scale applications.

The use of nanoparticles and surfactant foam has been shown to be a promising technique for the remediation of oil pollutants from soil. However, some nanoparticles, such as nZVI, nano-iron oxide, or silica nanoparticles, have been mainly used with surfactant foam. In the future, the combination of surfactant foam with other nanoparticles, such as Fe/Ni nanoparticles, Fe/Pd nanoparticles, or TiO_2_ nanoparticles, to remove oil-contaminated soil needs further research. Their useful lifetime will also need further evaluation.

Various soil types, for instance, sandy silt or fine sand, can be employed to investigate the efficiency of these approaches in a large-scale application. The treatment effectiveness of these methods under various environmental conditions, for example, pH or temperature, needs more research to determine their potential application range. Based on that, the optimal operating parameters on the lab and field scale can be obtained. The treatment efficiency and mechanisms of surfactant foam–nanoparticles on different oil compounds in soil needs more investigation, which will estimate the potential utilization of this mixture in a wide range of contaminated sites. In addition, more analysis on the cost and stabilization of surfactant foam and nanoparticles in the mixture is needed to apply this technique on the field scale.

The posttreatment processes also need further study. Oil pollutants require more treatment processes, such as bioremediation using oil-degrading bacteria, to be removed entirely. The evaluation of these bioremediation methods is required to optimize the treatment process.

The remediation rate of oil pollutants by nanoparticle-stabilized surfactant foam can be used to build a model to predict the fate and transport of oil pollutants in soil. However, the potential transformation of nanoparticles needs further investigation to provide more accurate data for the model. The effect of nanoparticle size on foam stability and quality, which will influence the remediation efficiency, also needs more study. Furthermore, it is critical to research the possible modification of the nanoparticle surface in the presence of surfactant foam, which will help to improve their stability and reactivity for the remediation of oil pollutants in soil.

The negative effect of nanoparticles on soil microorganisms and human health is controversial. Therefore, it is necessary to study on the potential toxicity of nanoparticles more, especially in a bench or pilot-scale environment, to evaluate the eco-safety of this method. More investigations on the in situ generation of surfactants, operation costs, recycling, and reuse of biosurfactants and nanoparticles are needed for full-scale application.

## Figures and Tables

**Table 1:** Use of selected nanoparticle for remediation of oil pollutants from soil.

Nanoparticle Name	Pollutant Name	Treatment Time, Day	Treatment Efficiency, %	Reference
MWCNTs ^1^	Phenanthrene	21	54.2	[61]
MWCNTs	PAHs ^2^	5	79	[62]
nZVI ^3^	Phenol	12 h	9	[63]
nZVI/BFN ^4^	Phenol	7 h	98.5	[63]
Iron nanoparticles	PCBs ^5^	6 h	95	[64]
nZVI	PAHs	1 h	70	[65]
APU nanoparticles ^6^	PAHs	5	67	[66]
nZVI/biosurfactant	Oil compounds	1 h	83	[40]
nZVI/biosurfactant foam	Oil compounds	30 min	67	[34]
nZVI	PCBs	15	42	[67]
nZVI-Pd	PCBs	15	64	[67]
nFe_3_O_4_	PCBs	15	68	[67]
Fe-Cu/biochar/geopolymer	Naphthalene	2 h	68	[68]
nZVI/bioattenuation	Diesel fuel	75	41.0	[21]
nZVI/biostimulation	Diesel fuel	45	64.6	[21]
nZVI/bioaugmentation	Diesel fuel	15–30	85.3	[21]
nZVI/biostimulation + bioaugmentation	Diesel fuel	30–60	89.5	[21]
Iron oxide nanoparticles	Crude oil	1	N/A	[42]
Nano rutile TiO_2_	Pyrene	25 h	52.2	[69]
Nano rutile TiO_2_	Phenanthrene	25 h	38.9	[69]
Iron oxide nanoparticles	PAHs	5	70	[70]
Akaganeite nano-rods	PAHs	1	65	[71]
Iron oxide nanoparticles	Anthracene	10	99	[41]
Graphene oxide	PAHs	7 min	~100	[72]
Fe-doped TiO_2_ nanocatalyst	PAHs	35 min	80	[73]
TiO_2_-based ZnHCF nanocomposite	PAHs	1	86	[74]
C_3_N_4_/Fe_3_O_4_ nanocomposite	Phenanthrene	2 h	92.3	[75]
Cu_2_OPLA composite nanofiber	Fluoranthene	8 h	67.6	[76]

^1^ MWCNTs = multiwalled carbon nanotubes. ^2^ PAHs = polycyclic aromatic hydrocarbons. ^3^ nZVI = nanoscale zerovalent iron. ^4^ BFN = *Bacillus fusiformis* bacterium. ^5^ PCBs = polychlorinated biphenyls. ^6^ APU = amphiphilic polyurethane.

**Table 2:** Use of some surfactants for the removal of oil pollutants in soil.

Surfactant Name	Surfactant Type	Oil Compound	Remediation Time, Hour	Remediation Efficiency, %	Reference
SDS ^1^	Anionic	Aliphatic	0.5	92	[90]
SDS	Anionic	Aromatic	0.5	77	[90]
Dodec-MNS	Anionic	TPH ^3^	0.8	68	[91]
SDS	Anionic	TPH	0.5	80	[85]
C_12_-MADS	Anionic	PAHs ^4^	72	68	[24]
SDBS ^2^	Anionic	PAHs	72	54	[24]
Tween 20	Nonionic	TPH	0.8	48	[91]
Triton X-100	Nonionic	PAHs	72	38	[24]
Span 20 + Tween 80	Nonionic	Diesel	1	48	[27]
Tween 80	Nonionic	TPH	24	40	[92]
Triton X-100	Nonionic	TPH	24	35	[92]
Rhamnolipid	Biosurfactant	TPH	24	63	[92]
Surfactin	Biosurfactant	TPH	24	62	[92]
Rhamnolipid	Biosurfactant	TPH	0.5	78	[85]
Saponin	Biosurfactant	TPH	0.5	76	[85]
Rhamnolipid	Biosurfactant	TPH	0.5	59	[93]

^1^ SDS = sodium dodecyl sulfate. ^2^ SDBS = sodium dodecylbenzene sulfonate. ^3^ TPH = total petroleum hydrocarbons. ^4^ PAHs = polycyclic aromatic hydrocarbons.

**Table 3:** Degradability of some surfactants.

Surfactant Name	Chemical Formula	Degradability, %	Degradation Time, Days	Reference
AOS ^1^	C_16_H_31_SO_3_Na	99	3	[99]
AOT ^2^	C_20_H_37_SO_7_Na	90	7	[100]
CTAB ^3^	C_19_H_42_BrN	98	13	[101]
JBR425 ^4^	C_32_H_58_O_13_	92	7	[102]
LAS ^5^	C_18_H_29_SO_3_Na	99 (under aerobic condition)	N/A ^9^	[103]
SAP ^6^	C_36_H_58_O_9_	93	3	[104]
SDBS ^7^	C_12_H_25_C_6_H_4_SO_3_Na	20	N/A	[105]
SDS ^8^	NaC_12_H_25_SO_4_	100	N/A	[106]
Steol	CH_3_(CH_2_)_10_CH_2_-(OCH_2_CH_2_)_n_OSO_3_Na	100	6	[100]
Triton SP	C_14_H_22_O(C_2_H_4_O)n	90	1.3	[107]
Tween 20	C_58_H_114_O_26_	20 (under anaerobic condition)	N/A	[108]
Tween 40	C_62_H_125_O_26_	20	28	[109]
Tween 80	C_64_H_124_O_26_	99	0.3	[26]

^1^ AOS = alpha olefin sulfonate. ^2^ AOT = sodium dioctyl sulfosuccinate. ^3^ CTAB = cetrimonium bromide. ^4^ JBR425 = rhamnolipid biosurfactant. ^5^ LAS = linear alkyl benzene sulfonate. ^6^ SAP = saponin. ^7^ SDBS = sodium dodecylbenzenesulfonate. ^8^ SDS = sodium dodecyl sulfate. ^9^ N/A = not available.

**Table 4:** Use of anionic surfactant foam for treatment of oil from contaminated soil.

Surfactant Name	Foam Type	Foam Concentration, mg/L	Pollutant Type	Treatment Efficiency, %	Reference
SDS ^1^	Spraying foam	6.6	Diesel	73.7	[20]
AOS ^2^	Spraying foam	6.6	Diesel	71.8	[20]
LAS ^3^	Spraying foam	6.6	Diesel	65.9	[20]
Steol CS-330	Microfoam	1800	TCE ^5^	75	[132]
SDS	Microfoam	2300	Diesel	62.9	[134]
RML ^4^	Microfoam	100	Diesel	44.75	[134]
SDS	Foam flushing	5000	PCBs ^6^	75.8	[135]
SDS	Foam flushing	50,000	DNAPL ^7^	93–97	[131]
SDS	Microfoam	2300–11,700	Diesel	88	[133]
Standapol ES-2	Foam flushing	1000	PAHs ^8^	N/A ^10^	[116]
GL5757	Foam flushing	2000	TCE	60	[137]
RML	Foam flushing	1%	PCP ^9^	67	[123]

^1^ SDS = sodium dodecyl sulfate. ^2^ AOS = alpha olefin sulfonate. ^3^ LAS = linear alkyl-benzene sulfonic-acid. ^4^ RML = rhamnolipid biosurfactant. ^5^ TCE = trichloroethylene. ^6^ PCBs = polychlorinated biphenyl. ^7^ DNAPL = dense nonaqueous phase liquid. ^8^ PAHs = polycyclic aromatic hydrocarbons. ^9^ PCP = pentachlorophenol. ^10^ N/A = not available.

**Table 5:** Use of some surfactant foams and nanoparticles to remove oil pollutants from soil.

Surfactant Foam Name	Nanoparticle Type	Pollutant Type	Treatment Efficiency, %	Reference
RML ^1^	nZVI	Crude oil	67	[34]
APG-Ph ^2^	nZVI	Diesel	95	[86]
APG-Ph	Fe_3_O_4_	Diesel	76	[86]
SDS ^3^	SiO_2_	Crude oil	68	[118]
SLS ^4^	SiO_2_	Diesel	95	[169]
Tween 20	SiO_2_	Diesel	78	[170]
APG-Ph	SiO_2_	Diesel	54	[171]
APG-Ph	nZVI	Diesel	98	[172]
SDS	SiO_2_	Crude oil	54	[173]
Tween 80	Ni^0^	Diesel	99	[174]
Tween 80	Cu^0^	Diesel	99	[174]
RML	Zn/Fe^0^	Diesel	84	[175]

^1^ RML = rhamnolipid biosurfactant. ^2^ APG-Ph = alkyl polyglucoside phosphate. ^3^ SDS = sodium dodecyl sulfate. ^4^ SLS = sodium lauryl sulfate.

## Data Availability

The data presented in this study are available on request from the corresponding author.
